# Spatially Estimating Disturbance of Harbor Seals (*Phoca vitulina*)

**DOI:** 10.1371/journal.pone.0129798

**Published:** 2015-07-01

**Authors:** John K. Jansen, Gavin M. Brady, Jay M. Ver Hoef, Peter L. Boveng

**Affiliations:** National Marine Mammal Laboratory, Alaska Fisheries Science Center, National Oceanic and Atmospheric Administration, Seattle, Washington, United States of America; Sonoma State University, UNITED STATES

## Abstract

Tidewater glacial fjords in Alaska provide habitat for some of the largest aggregations of harbor seals (*Phoca vitulina*), with calved ice serving as platforms for birthing and nursing pups, molting, and resting. These fjords have also been popular destinations for tour ships for more than a century, with dramatic increases in vessel traffic since the 1980s. Seals on ice are known to flush into the water when approached by tour ships, but estimating the exposure to disturbance across populations is difficult. Using aerial transect sampling while simultaneously tracking vessel movements, we estimated the spatial overlap between seals on ice and cruise ships in Disenchantment Bay, Alaska, USA. By integrating previously estimated rates of disturbance as a function of distance with an ‘intensity surface’ modeled spatially from seal locations in the surveys, we calculated probabilities of seals flushing during three separate ship visits. By combining our estimate of seals flushed with a modeled estimate of the total fjord population, we predict that up to 14% of the seals (up to 11% of pups) hauled out would have flushed into the water, depending on the route taken by ships relative to seal aggregations. Such high potential for broad-scale disturbance by single vessels (when up to 4 ships visit per day) was unexpected and underscores the need to 1) better understand long-term effects of disturbance; 2) regularly monitor populations exposed to high vessel traffic; and 3) develop conservation measures to reduce seal-ship overlap.

## Introduction

Marine mammals are increasingly exposed to perturbations by humans in their habitats; in the water, on shore, and on ice. With increasing human presence on the coasts and in the oceans, long-term population effects on marine mammals are increasingly likely and, in many species, already documented (e.g., bottlenose dolphin *Tursiops truncatus*, beluga whale *Delphinapterus leucas*, California sea lion *Zalophus californianus*, and Hawaiian monk seal *Monachus schauinslandi*: [1–5]. Even though findings of human disturbance on wildlife are mounting, most are based on behavioral responses so that predictions of broader-scale impacts are sometimes viewed as speculative and lacking a basis for real concern. It is still widely debated whether reactions to disturbance, even those linked to altered habitat use (e.g., a local decline in abundance) or physiology (e.g., elevated stress hormones), are biologically meaningful. Such findings coupled with modeling of likely effects (e.g., energetic costs) make compelling arguments for population-level effects, yet managers are often hampered by circumstantial nature of the evidence when seeking support for conservation actions. We believe that in the vast majority of human-marine mammal interactions a central question persists: what is the risk that impairment of individual fitness, especially when it appears nominal, can alter the dynamics of a population and over time threaten its persistence? In other words, how many effects on individuals compose an impact to the population?

The often transient nature and large ranges of marine mammals complicate even basic assessment of impacts because perturbations and their effects may be spread unpredictably across a population. So deriving and ultimately tracking measures of population-wide exposure to disturbance is difficult but nevertheless critical for understanding the cumulative and chronic threats to a population. When estimation of such measures has been possible, such as with precise, long-term measurements of human-wildlife interactions, impacts of disturbance have been surprisingly complex, even cascading from populations to the community level [6]. In our study, we developed a new technique for approximating vessel disturbance across a local population of harbor seals in a glacial fjord.

Harbor seals have one of the widest ranges of pinnipeds, with five subspecies inhabiting coastal areas bordering the North Pacific and North Atlantic oceans from the Arctic (78°N) south to the Tropic of Cancer (20°N). Despite the species’ diverse marine and terrestrial habitats, individual harbor seals exhibit fidelity to specific haul-out areas used for breeding and molting [7–12]. Fidelity to such sites may put them at greater risk from disturbance. When confronted with a stressor, the reluctance of wildlife to move away (i.e., tolerance) is theorized to reflect, at least partly, the importance of that particular area for life processes, rather than indifference to the disturbance, with animals managing the consequences of staying rather than those of leaving [13–16]. For instance, a severely depleted population of beluga whales contracted its range to nearly one-third, an area thought to provide a balance between prey availability and predator avoidance, but thereby subsequently occupied a core area most impacted by industrial growth and vessel traffic [2]. Similarly, harbor seals in an urbanized estuary used areas with high seasonal abundance of prey despite these areas coinciding with high levels of disturbance, an effect attributed to seals tolerating and/or habituating to human presence to optimize feeding opportunities [16].

The largest aggregations of harbor seals in the world use floating ice in glacier fjords of Alaska, USA, as a platform on which to whelp and nurse pups and rest while molting (e.g., >5400 animals at Icy Bay, Alaska)[17]. These sites have experienced a ten-fold increase in tour ship visitation since the 1980s, leading to concern over population impacts to harbor seals. Despite their increasing presence, visitation by tour ships at four of the five most popular sites–Tracy Arm (268 ship visits in 2013), Endicott Arm (38), College Fjord (41), and Disenchantment Bay (143) is unregulated and existing guidelines do little to moderate chronic disturbance [18–20]. At Glacier Bay National Park, Alaska, considered one of the most sought-after cruise destinations (233 annual cruise ship visits), long-standing regulations under a special Park authority preclude ships from entering seal haul-out areas during the critical pupping period, with no more than 2 cruise ships permitted throughout the bay on a given day (U.S. Code of Federal Regulations; 36 CFR 13.65).

Harbor seals flush from floating glacial ice with increasing frequency as cruise ships approach closer than 400 to 500 m, with evidence that seals flush at up to 1000 m [17, 20, 21]. In Disenchantment Bay, Alaska, despite regular flushing, as ship presence increased, seals seemingly did not leave the area or actively avoid ice drifting in areas used by ships [22]. But this disturbed population had a lower proportion of pups and contrasting seasonal trends compared to an adjacent, undisturbed glacial fjord [17, 22]. Chronic flushing by vessels would likely increase the time seals are submerged in the water. Physiological models suggest this could disrupt energy balance, particularly for young pups, and compromise growth and survival [20, 23]. Regular and unmonitored disturbance combined with evidence of differences in how seals use disturbed vs. undisturbed habitat increases concern that ship presence could be altering population functions (i.e., vital rates) which, being difficult to measure, could go unnoticed for some time before a decline was detected.

To gauge the potential for chronic, widespread disturbance, we sought to quantify the effects that occur across all seals hauled out in a fjord during a typical cruise ship visit; that is, what proportion of the population reacts by flushing into the water. We estimate the total number (and proportion) of seals flushing into the water during cruise ship visits, by examining the spatial and temporal overlap between seals and ships.

## Materials and Methods

### Ethics Statement

This research was conducted under the Marine Mammal Protection Act Scientific Research Permit 782–1676 issued to the National Marine Mammal Laboratory by NOAA's Protected Resources Division.

### Aerial surveys

**Fig 1:**
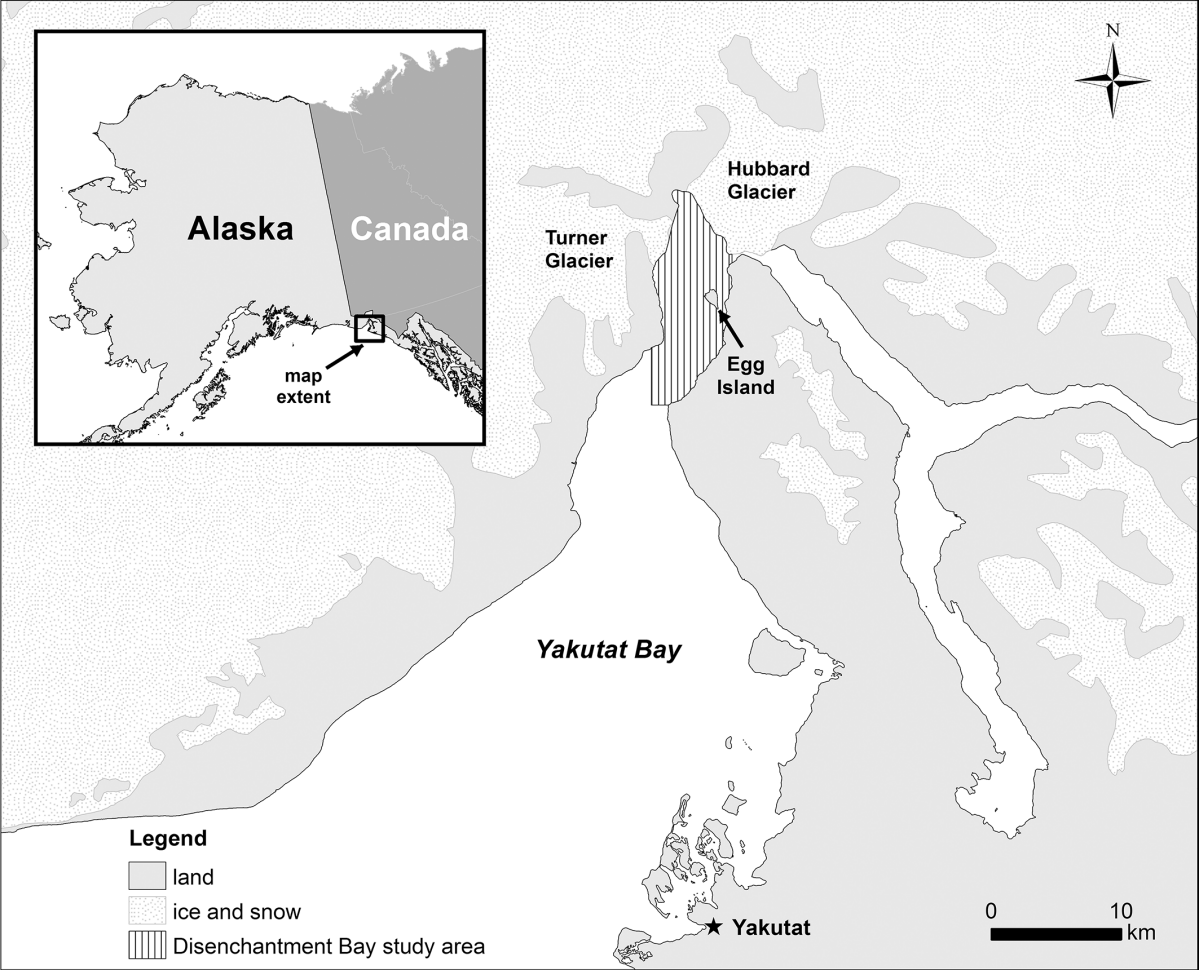
Map of Disenchantment Bay study area near Yakutat, Alaska, USA. The extent of snow and ice-covered terrain (stippled area) was derived from a NOAA Coastal Service satellite photo taken in 1993.

Aerial surveys were conducted to map the distributions of seals as part of a larger study of the longer-term response of seals to cruise ships in Disenchantment Bay, near Yakutat, Alaska (Fig 1). On three occasions, our aerial surveys overlapped or closely preceded a cruise ship visit to the study area, one in each of the months of May (survey A), June (B), and August (C) (Table 1). Surveys A and B occurred roughly when we expected to find peak numbers of seal mothers nursing pups, and survey C occurred near the midpoint in the seal molting period. Seal distributions were mapped via aerial vertical photography, and cruise ship tracks were recorded with a GPS (Garmin 76S; <5 m accuracy) by an onboard observer. Having close spatial and temporal correspondence provided a virtual snapshot of the distribution of seals that the ships would encounter traveling in the bay. Each snapshot included an aerial transect survey of 70 km^2^ of floating ice habitat completed in about 1 h followed immediately by a cruise ship completing its transit in and/or out of the fjord in about 2–3 h. Despite the time elapsed between the survey and ship completing its transit, when tide and wind currents were moving the ice, previous surveys have shown that general distributional patterns persist even across subsequent days adding support for this method of estimating broad-scale disturbance [22].

**Table 1:** Timing of aerial surveys of harbor seals in relation to visits by cruise ships in Disenchantment Bay, Alaska, USA.

Survey	Cruise ship leg	Date	Time of aerial survey(Alaska Daylight Time)		Time of ship visit within survey area (h)			Total Time Elapsed (h:m)
			Start (h)	End (h)	Enter (h)	Turn-around (h)	Exit (h)	
A	Out	31 May 2005	1240	1419	1241	1434	1524	2:44
B	In/Out	22 Jun 2004	1131	1257	1245	1440	1547	4:16
C	In/Out	25 Aug 2005	1249	1426	1250	1426	1506	2:17

Surveys were selected to estimate disturbance based on a close temporal correspondence with a ship entering the study area. For survey A, the ship had already begun its inbound leg when surveys began and so disturbance was estimated just during the outbound leg.

Aerial surveys were flown over the ice haul-out area used by harbor seals in Disenchantment Bay in 2004 and 2005 in the early afternoon when seals typically haul out in peak numbers (Table 1) [17, 24]. The survey aircraft, a single-engine DHC Beaver, was flown at 1000 ft and *ca*. 100 knots on preset transects spaced either 400 m (in 2004) or 200 m (in 2005) apart and oriented approximately north-south from the mouth of the bay (north of Pt. LaTouche) to the face of Hubbard glacier (Fig 1). A vertically-mounted camera (Nikon D1X with a 60 mm lens) captured an image every 2 seconds through a portal, each image covering about 80 X 120 m at the surface of the water. This image capture rate, and the spacing of the transects, allowed for a gap between images of about 30 m end-to-end and 280 m (in 2004) or 80 m (in 2005) side-to-side, thus ensuring that images were independent from one another; that is, seals were sampled only once. About 1500–2000 images were taken during each survey representing 20–25% coverage by area of the bay. The camera was usually turned off when flying over large areas of open water where hauled out seals would be absent. Our focus was the predicted frequency of seals flushing from the ice into the water, but we do not discount other impacts from vessel presence or noise on seals resting on the ice or swimming/diving in the water.

### GIS Analyses

Images were georeferenced and embedded as a raster layer in an ArcGIS (Version 9.3, Esri Inc., Redlands, CA) project. Seals were spatially marked and counted in a point layer. Footprints showing the extent of each image were generated as polygons in a separate layer and seal points were summed, spatially assigned to image centroids, and exported for statistical analysis. The spatial extent of each image was assumed to be constant despite random variation in altitude (max: ± 30 m) during the survey. The occurrence of seals in areas not covered by photographs was approximated using spatial statistical techniques (see below) that derived interpolated densities over a regularly spaced grid (0.45 km^2^ cells) corresponding to the study area. The grid size was chosen to balance the time needed for computations with the ability to derive a representative density surface. Each grid cell’s density estimate was assigned to a point at its centroid. The total spatial extent of each day’s survey effort–over which the interpolated density grid was calculated–was delineated by creating a polygon that was bounded by: 1) the coastline of the bay (east and west shorelines from line shapefiles provided by the Alaska State Department of Natural Resources [DNR]); 2) an estimate of the location of the face of the glacier (by connecting points that marked the glacial terminus in each of the northernmost images from every transect; and 3) the southern extent of the ice field defined as the edge of the southernmost image in each transect where ice suitable for hauling out (≥ 2 m at longest axis) was present by visual estimation. Areas of open water (i.e., without suitable ice) of ≥ 1 km^2^ within the ice field were delineated by “donut-holes” in the overall polygon where the spatial boundaries were defined by the outermost images in which suitable ice was absent. For graphical display, the interpolated density estimates were smoothed using the kernel density tool in the spatial analyst extension of ArcMap (Version 10.0, Esri Inc., Redland, CA) using a 400 m search radius and default settings for the remaining parameters.

### Spatial Statistics

Spatial statistical methods were used to create an intensity surface from sampled seal densities [25]. This surface, or intensity function, is a 2-dimensional probability density function on the x- and y-coordinates (contained in the vector *s*) over a region, where the probability is scaled so that it also carries information on relative abundance. This produces a model of the expected number of seals in any given region. If we define this surface as *λ*(*s* | *θ*), where the function depends on parameters *θ*, then for an inhomogeneous Poisson process the count over any region *A* is distributed as a Poisson random variable with mean *μ*(*A*) = ∫_*A*_ *λ*(*s* | *θ*)*ds* [26]. In other words, from an estimated intensity function we can obtain an estimate of the expected number of seals for any region *A* by using *μ*(*A*). Estimating *λ*(*s* | *θ*) generally involves estimating the parameters *θ* that control its shape. The method of Ver Hoef and Jansen [25] for estimating *λ*(*s* | *θ*) from aerial photographs was used to account for their non-random placement along transects. We can also obtain the variance of *μ*(*A*) = ∫_*A*_ *λ*(*s* | *θ*)*ds* for any region *A*. In practice, the intensity surface is approximated as a density surface by using a fine grid of systematic polygons (generally squares, such as in a clipped raster grid), where the value in each grid cell is the expected number of animals divided by the area of that cell. We will call this the estimated density grid.

### Disturbance Estimation

The closest approach distance of a cruise ship to each seal’s location was used to assign a probability of that seal flushing into the water. These probabilities were based on observations from cruise ships in Disenchantment Bay from Jansen et al. [20] that established the relationship (in 100 m increments) between ship-to-seal distance and the probability of flushing. These results showed that seals begin flushing at a minimum of 400 m with an average 89% of seals flushing when ships approached ≤ 100 m [20]. The probabilities of known seals (i.e., mapped during surveys) flushing that were within 400 m of cruise ship tracks were summed to derive an estimate of the total number of seals flushed based on ship distance at closest approach (e.g., two seals with a 50% probability of flushing = one seal predicted to flush). We also calculated closest approach distances to the interpolated grid centroids (i.e., seal density points) and for those ≤ 400 m assigned each a flushing probability. We then multiplied each centroid’s density estimate (# of seals per km^2^) by its flushing probability and the area of the cell (subtracting area covered by image footprints) to arrive at an estimated number of seals flushing per cell. Summing this value for all cells ≤ 400 m from the ship track provided an estimate of the number of (‘interpolated’) seals flushing in areas not covered by the photographs. Adding the number of surveyed and interpolated seals predicted to flush provided an estimate of the total number of seals that flushed during each ship’s transit of the bay.

Total abundance in the study area was estimated by averaging all the individual grid-cell density estimates (# of seals per km^2^) and multiplying by the total study area (subtracting the image footprints), and then summing the number of interpolated and surveyed seals. The proportions of seals disturbed were then calculated as: (total number of seals disturbed) ÷ (total abundance). We assessed the above measures of risk for pups separately because there is evidence that they may be more prone to effects of flushing, particularly via disruptions to nursing and energy balance caused by increased time submerged in the near-freezing water of our study area [20].

## Results and Discussion

**Fig 2:**
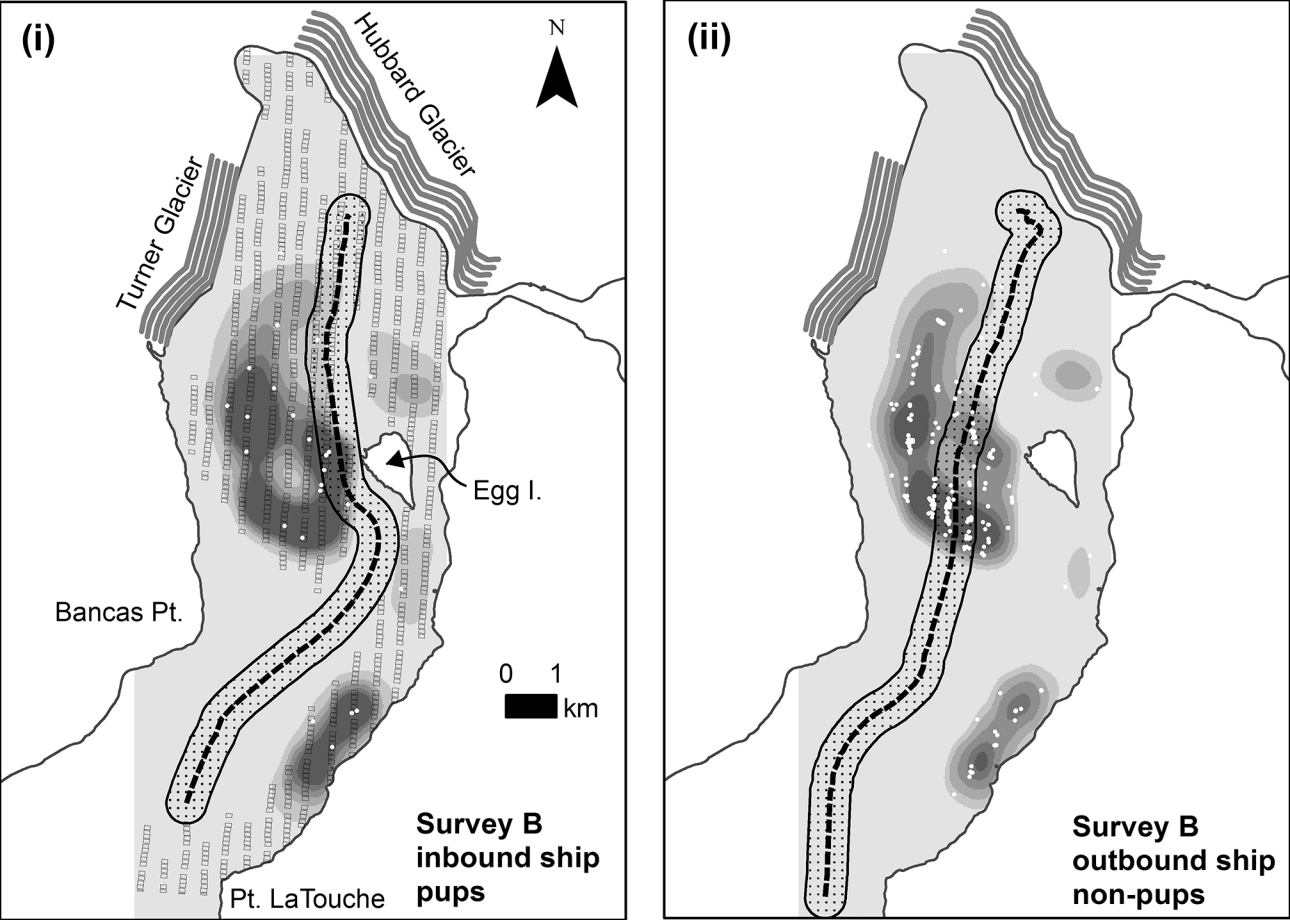
Locations and predicted densities of harbor seals from aerial survey on 22 June 2004 in Disenchantment Bay, Alaska, USA. For clarity and to reduce clutter on the map, densities of pups are shown in panel (i) and non-pups in panel (ii). The inbound and outbound legs of the cruise ship visit are shown in panels (i) and (ii), respectively. Seal densities are on a gray scale from low (light gray) to high (black). The small black rectangles in (i) illustrate the surface coverage of geo-referenced aerial images taken during transects over the seal haul-out area when there was ≥5% ice coverage. The dotted line shows the route of the cruise ship with the stippled band representing the area within 400 m where seals are more likely to be disturbed and flush into the water (see text). The white dots mark actual locations of seals identified from the aerial images. Pupping phenology follows Jansen et al. [22]. For graphical display, seal densities estimated at grid-cell centroids were smoothed using the kernel density tool in the spatial analyst extension of ArcMap (Version 10.0, Esri Inc., Redlands, CA) (see text).

The level of disturbance we predicted varied with factors related to both seals and ships. The highest levels of disturbance (216 seals; 14%) occurred following survey B (June) on the ship’s outbound transit, coincident with the highest seal abundance we observed in the three surveys (Table 2, Fig 2). Ice cover in Disenchantment Bay typically peaks in June providing the largest area of haul-out habitat at a time when the peak number of mothers are rearing pups [22]. Cruise ship traffic reached its highest levels by early June, with as many as 4 ships visiting per day, and continued at high levels through August. Near-peak levels of ice generally persist until mid-July, creating navigational challenges for ships as they usually attempt to follow open leads in order to avoid larger pieces of ice and maintain higher speeds. For this reason, ships sometimes entered the bay northbound favoring the eastern coast south of Egg Island, where open water often occurred due to shielding from southward drifting ice (Fig 1) [22]. For survey B, the ship avoided the densest area of seals (and ice) on its inbound route, resulting in a relatively low predicted overall disturbance (55 seals; 4%) but a somewhat higher proportion of disturbed pups (36 pups; 7%). It is unclear why the ship took a different outbound route, traversing the densest aggregation of seals and thereby increasing predicted disturbance almost 4-fold (Fig 2). The route was a more direct exit from the bay but we expect the ship would have encountered more ice and had to maintain lower speeds. More direct outbound routes (compared to inbound) appear to be common, occurring in nearly 40% of the ship courses that we tracked venturing north of Egg Island; only one ship had a more direct inbound route (N = 34 ships tracks in 2004; 31 in 2005). It may be that more meandering routes inbound occur because the structured tour of the bay typically takes place at this time, when a presentation about the area is broadcast over the public address system. The presentation usually ends at about the time the ship stops closest to the glacier, rotates, and turns outbound. So, when ships begin their exit and most passengers have left the outer decks, priorities may shift to transiting to the next destination as soon as possible using the shortest route possible. Whatever the motivation, ships following a route in the center of the bay, when ice is present there, would on average be expected to flush more seals than those following a route 1–2 km from the eastern shoreline. As noted in Jansen et al. [20], without a trained observer on the ship’s bridge, it is difficult to detect seals at a distance where maneuvers for avoidance are still possible, suggesting that ship personnel are largely unaware of and thus unresponsive to the distribution of seals. Our sample of ship tracks suggests that through careful route selection the incidence of flushing can be reduced substantially.

**Table 2:** Number (rounded) and proportion of harbor seals predicted to flush into the water in response to an approaching cruise ship in Disenchantment Bay, Alaska, USA.

Survey	Phenology	Cruise Ship Leg	No. of seals predicted to flush (% of total abundance)	No. of pups predicted to flush (% of total pups)	Total estimated abundance (± SE)
A	early-pupping	Out	19 (1)	8 (2)	1370 (100)
B	mid-pupping	In	55 (4)	36 (7)	1525 (152)
		Out	216 (14)	56 (11)	
C	molting	In	6 (1)	na	624 (52)
		Out	0	na	

Ship visits coincided with three aerial surveys, A-C. Pups were identified based on a discriminant analysis using body length measurements from aerial vertical imagery of known pups (i.e., suckling) corrected for measured growth rate (unpublished data^1^), combined with standard body lengths of known-age yearlings at other glacial sites^2^. Pups were not distinguishable during molting.

^1^Data analysis and results available online at ftp://ftp.afsc.noaa.gov/posters/pJansen06_productivity-pup-growth.pdf.

^2^ Unpublished data from G. Blundell, Department of Wildlife Conservation, Alaska Department of Fish and Game, P.O. Box 110024, Juneau, AK. 99811 USA.

**Fig 3:**
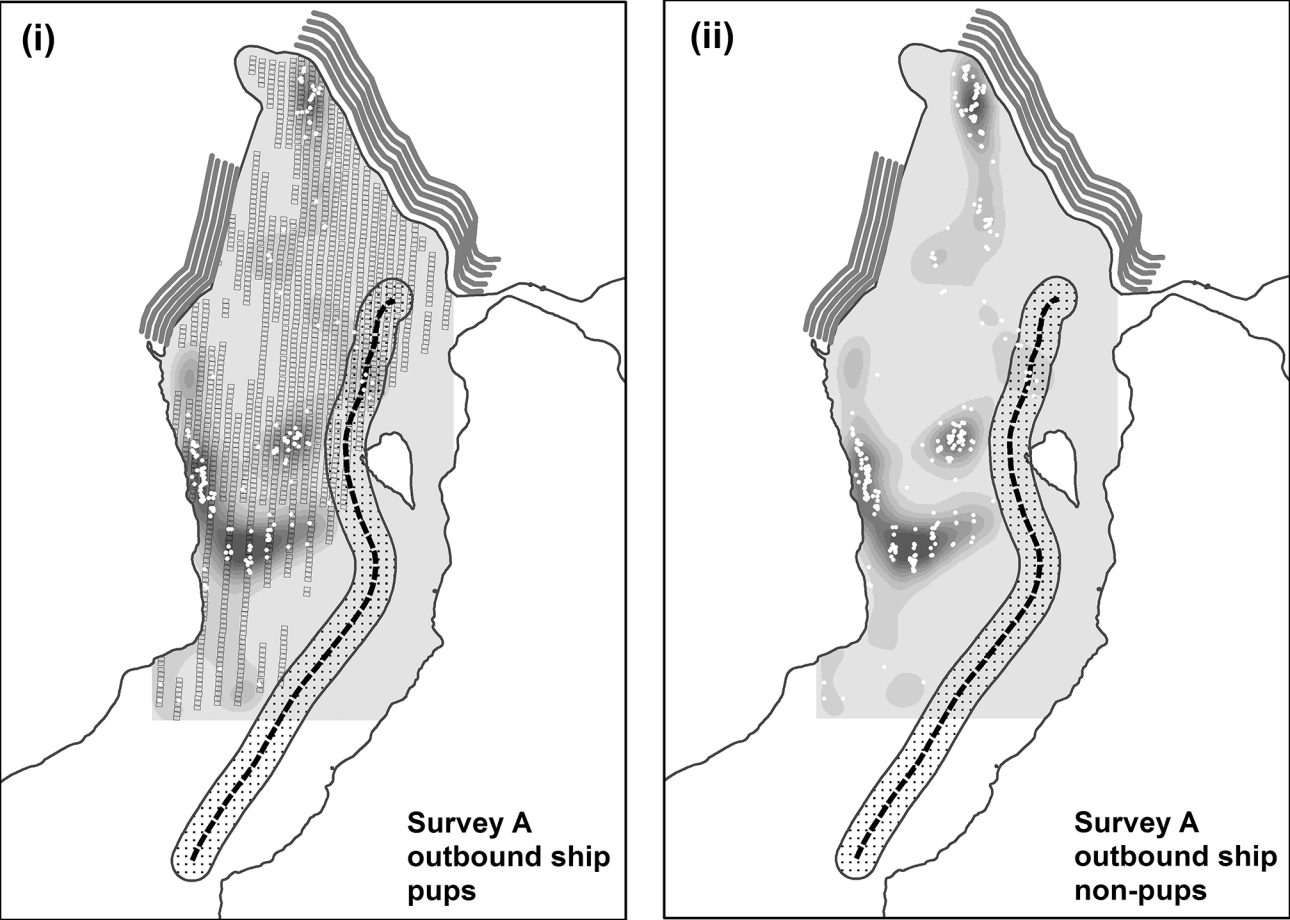
Locations and predicted densities of harbor seals from aerial survey on 31 May 2005 in Disenchantment Bay, Alaska, USA. For clarity and to reduce clutter on the map, densities of pups are shown in panel (i) and non-pups in panel (ii). See Fig 2 for geographical references. Seal densities are on a gray scale from low (light gray) to high (black). The small black rectangles in (i) illustrate the surface coverage of geo-referenced aerial images taken during transects over the seal haul-out area when there was ≥5% ice coverage. The outbound leg of the cruise ship is shown in both panels. The dotted line shows the route of the cruise ship with the stippled band representing the area within 400 m where seals are more likely to be disturbed and flush into the water (see text). The white dots mark actual locations of seals identified from the aerial images. Pupping phenology follows Jansen et al. [22]. For graphical display, seal densities estimated at grid-cell centroids were smoothed using the kernel density tool in the spatial analyst extension of ArcMap (Version 10.0, Esri Inc., Redlands, CA)(see text).

**Fig 4:**
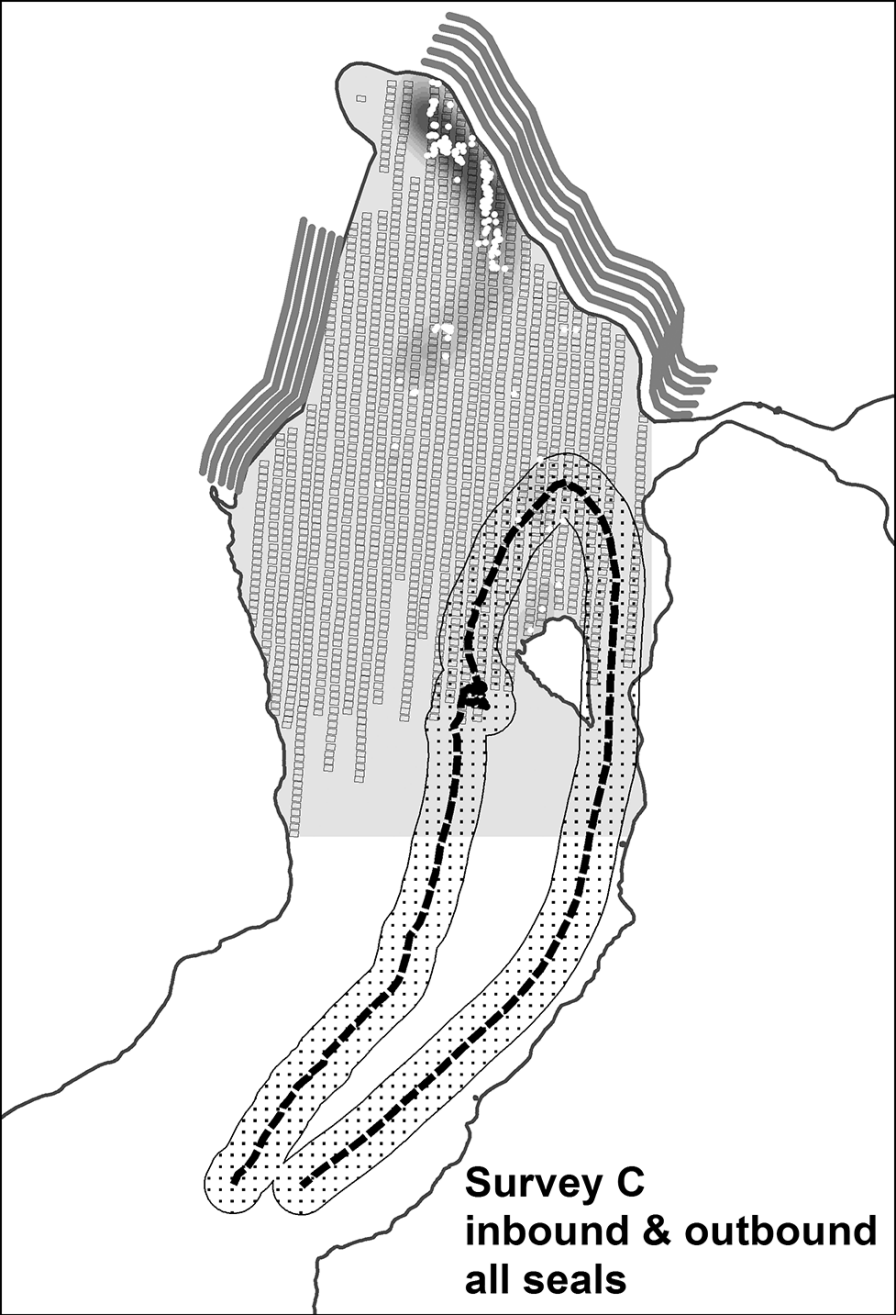
Locations and predicted densities of harbor seals from aerial survey on 25 Aug 2005 in Disenchantment Bay, Alaska, USA. All seals are shown because pups are not distinguishable in August. See Fig 2 for geographical references. Seal densities are on a gray scale from low (light gray) to high (black). The small black rectangles illustrate the surface coverage of geo-referenced aerial images taken during transects over the seal haul-out area when there was ≥5% ice coverage. The entire ship track is shown with the ship stopping at the northernmost point and then exiting closest to the eastern shoreline. The dotted line shows the route of the cruise ship with the stippled band representing the area within 400 m where seals are more likely to be disturbed and flush into the water (see text). The white dots mark actual locations of seals identified from the aerial images. Pupping phenology follows Jansen et al. [22]. For graphical display, seal densities estimated at grid-cell centroids were smoothed using the kernel density tool in the spatial analyst extension of ArcMap (Version 10.0, Esri Inc., Redlands, CA)(see text).

Disturbance was minimal during the other two ship routes we examined. Despite relatively high numbers of seals present (and peak numbers of pups), the vessel we tracked outbound following survey A was predicted to stay > 400 m from the vast majority of seals (Fig 3). By transiting closer to Egg Island and the eastern shoreline, similar to the inbound route following survey B, this vessel was predicted to flush only 19 seals; 1% overall and only 2% of pups present (Table 2). The vessel we tracked late in the season during molting (following survey C) and at the seasonal low in abundance had the lowest predicted disturbance (6 seals; 1%; no pups; Table 2). Here, the distribution of seals was largely north of Egg Island but still within the normal transit corridor used by cruise ships late in the season when reduced ice allows close views of Hubbard glacier to within 1 km [22]. This ship not only turned around well south of Hubbard glacier, avoiding a close approach of the seals, but further minimized disturbance by selecting an exit route between Egg Island and the eastern shoreline (Fig 4). An exit via this channel was used by only 11% of the vessels we tracked despite typically low ice density east of Egg Island. [22].

It’s not surprising that we found the highest disturbance when the seasonal occurrence of both seals and ships were near peak levels, combined with a ship selecting a route through the densest ice at a time of day when the number of seals on the ice was likely near maximum. The timing of the ship visit in the early afternoon, when most ships visit Disenchantment Bay, coincides with what other studies (and our observations in the study area) have shown is the daily peak haul-out period for seals (i.e., 1200–1600 h, Alaska Daylight Time)[17, 24]. Close temporal overlap between ships and hauled out seals would be expected to increase the level of flushing disturbance due to vessels. Further, we believe our estimate of disturbance represents a potentially small fraction of what occurred over a longer interval, as the ship visit following survey B was the third to occur on that day. This visit was also at the end of an unusually active phase for ship traffic, being the fifteenth ship visit over the preceding 7 days. Moreover, we based our disturbance estimates on observations from ships which are necessarily limited in range; observations from elevated shore stations show that flushing of seals in response to cruise ships occur out at far as 1 km [21]. If there was variation in tolerance to ships, with some seals opting to move to less disturbed ice habitat prior to our surveys (e.g., Icy Bay, 115 km to the west), or if habituation to vessel presence exists, the seals mapped in our study (and those from which flushing distances were derived) would be disproportionately tolerant and our estimates of disturbance would be conservative compared to the expected response of seals in glacial fjords with zero or low vessel traffic.

Despite our largely opportunistic approach, which resulted in a small sample, our study demonstrates that integrating fine- (behavioral response) and course-scale data (distribution) is useful for assessing disturbance across larger spatial scales. Future studies that can better coordinate aerial surveys with detailed tracks of ships, and especially in conjunction with individual seal movements, would be an important step toward understanding the potentially compounding effects of repeated disturbance within and across days. Such studies examining individuals’ behavior should consider the statistical power needed to detect proximate differences in responses to vessels (e.g., tolerance or sensitivity) as well as changes that may occur as exposure to disturbance by tagged seals accumulates over one or more seasons. At Disenchantment Bay, some local Tlingit tribe members believe there has been a gradual decline in the seal population, perceived to have been caused by increasing exposure to disturbance (especially to mothers and pups) stemming from a ten-fold increase in cruise ship visitation over the last three decades [17]. If a decline occurred, or a change in distribution or availability of seals, multiple factors could have contributed including changes in glacier-dependent haul-out habitat, local and large-scale oceanography, predation, and broader human impacts. Given the history over the past century of tour vessels visiting glacial fjords, researchers designing new studies should consider that habituation or selection of seals more tolerant of ships may have occurred, and that responses of seals may be influenced by a combination of the population’s long-term exposure and seals’ individual relatively short-term experience with tour ships.

## Conclusions

Mounting vessel traffic in habitats of marine mammals in Alaska and the Arctic is cause for concern [27]. The world’s commercial fleet has tripled in the past 50 years and will increasingly have access to high latitudes and passages less restricted by sea ice [27, 28]. Cruise ship tourism in Alaska is following the same trend. Despite declines from the historic peak from 2007 to 2009, with over 1 million passengers in each year, cruises continue to dominate Alaska tourism with about 60% of the market [29]. With visitor trends returning to positive in 2011, and growth in 2012 back to pre-recession levels (6%), the global popularity of Alaska is now second only to the Caribbean as a desired cruise destination [30]. Despite fluctuations in the market, tidewater glaciers remain a central attraction for all cruise ships in Alaska waters–amounting to approximately 500 ship visits annually to fjords where neither restrictions nor appropriate vessel approach guidelines are in effect to minimize harbor seal disturbance [18, 20].

Findings that better define the overall exposure of wildlife populations to human disturbance, especially if there are uneven risks across age or sex classes, will guide future studies examining the linkages between altered behavior or physiology and individual survival and population health. For harbor seals in glacial fjords, identifying and quantifying these linkages will be challenging, almost certainly requiring multi-year, longitudinal studies of individuals under varying ship regimes and environmental conditions. Only under these controls can differences in tolerance to ships across a local population of seals be distinguished from individual seals’ processes of habituation. Until these studies are undertaken, we believe the awareness that a single visit by a ship to a fjord has the potential to disturb a considerable portion of the seals present, and that this disturbance can be mitigated through choice of a vessel’s route and timing, will aid managers in finding a balance between tourism and resource protection. The need for balance is particularly acute at tidewater-glacier fjords in Alaska where tour ship visits have been increasing for decades, where chronic disruptions of the seals’ natural behaviors have been documented, and where current wildlife viewing guidelines provide little protection for seals against vessel disturbance.

## Supporting Information

S1 Dataset Predictions of pup and non-pup seal densites (no. seals per km^2^) at Disenchantment Bay, AK., for three aerial surveys: A) 31 May 2005, B) 22 June 2004, and C) 25 Aug, 2005, with coordinates (m) in Albers equal area (NAD83). Locations represent the centroids of regularly-spaced grid cells (0.45 km^2^).
(ZIP)

S2 Dataset Locations of seals (by group) at Disenchantment Bay, AK. mapped during three aerial surveys: A) 31 May 2005, B) 22 June 2004, and C) 25 Aug, 2005, with coordinates (m) in Albers equal area (NAD83). (ZIP)

S3 Dataset Location tracks of cruise ships that entered Disenchantment Bay, AK. following three aerial surveys: A) 31 May 2005, B) 22 June 2004, and C) 25 Aug, 2005, with latitude and longitude in Albers equal area (NAD83). (ZIP)
